# Aligning valid research outcomes with stakeholder values—what do they need for decision-making?

**DOI:** 10.3389/fvets.2024.1444023

**Published:** 2024-09-11

**Authors:** David G. Renter, Jan M. Sargeant, Annette M. O’Connor, Audrey Ruple

**Affiliations:** ^1^Center for Outcomes Research and Epidemiology, College of Veterinary Medicine, Kansas State University, Manhattan, KS, United States; ^2^Department of Population Medicine, Ontario Veterinary College, University of Guelph, Guelph, ON, Canada; ^3^Department of Large Animal Clinical Sciences, College of Veterinary Medicine, Michigan State University, East Lansing, MI, United States; ^4^Department of Population Health Sciences, VA-MD College of Veterinary Medicine, Virginia Tech, Blacksburg, VA, United States

**Keywords:** outcomes, veterinary, validity, research impact, outcomes research

## Abstract

This paper is derived from a presentation given by the first author at the 2024 Symposium for the Calvin Schwabe Award, presented to Dr. Jan Sargeant for Lifetime Achievement in Veterinary Epidemiology and Preventive Medicine. Researchers must work toward ensuring validity throughout the research process, but we also should ensure that our resulting outcomes are specified to appropriately inform and enable decision-making by the end-users. Given the scope and diversity of topics addressed by veterinary researchers, the potential beneficiaries or stakeholders of our research also varies. Stakeholders or end-users may include veterinary practitioners, other researchers, livestock owners, “pet parents,” government officials, corporate entities, or the general public in the case of public health or food security and safety issues. Current research in animal agriculture provides an opportunity to consider research outcomes in a sustainability framework which concurrently values social, economic, and environment impacts of animal health and management decisions. In companion animals, contemporary issues of affordability and access to care, quality of life, or compliance effects on efficacy, also extend the spectrum of relevant research outcomes. In these cases, traditional measures of animal health, such as morbidity, mortality, or weight gain, may not be the most relevant for the end-users. Furthermore, if studies are not designed and analyzed with well-defined primary outcomes that are informed by stakeholders’ values, but rather post-hoc considerations of these values are made based on indirect or surrogate measures, there is the potential to incorporate error and bias into our conclusions and the end-users’ decision-making processes.

## 1 Introduction

When decision-makers strive to be informed by evidence or science, results from research are an essential and foundational component of this process. Research is often performed to determine whether a factor(s)—which may be described as an exposure, treatment, risk factor, intervention, or independent variable—is associated with an outcome or dependent variable. The selection of an appropriate outcome(s) to compare among groups is critical to maximizing research value and relevance to the end-user or stakeholders (1).

In the context of evidence-based medicine, the end-user of veterinary research is typically considered a clinician; yet, in a broader context, veterinary researchers may consider the end-user or stakeholders of their research to also include livestock owners or care-takers, “pet parents,” other researchers, government agencies, private industry, or even society in general. Outcomes can be defined at both the conceptual and operational level (1). For example, “health” may be a conceptual outcome of interest, but operational outcomes, that can be measured to evaluate “health,” could be various measures of morbidity or mortality. In animal agriculture, the framework of sustainability provides three conceptual domains: environmental, economic, and societal; within each of these domains, measurable operational outcomes for research purposes can be defined. Similarly, conceptual outcomes such as access to care, quality of life, animal welfare/well-being, or food safety and security can be further refined into operational outcomes that can be measured and incorporated into research plans. Regardless of the domain, research outcomes should be valid and relevant to the end-users for the research to have value and to enable appropriate decision-making. Here and elsewhere, discussions of research methodologies, including study design, implementation, analysis, and reporting, typically focus on operational outcomes—the things that are measured and analyzed.

A common research purpose is to determine differences in outcomes attributed to an intervention, where an intervention can be defined as many things, such as dietary changes or supplementations, changes to husbandry or management practices, implementation of vaccinations, or use of pharmaceuticals (1). While observational study designs are common in veterinary research, randomized controlled trials are considered the strongest empirical research design for establishing that an observed difference in the outcome was due to the intervention (1, 2). Thus, the concepts discussed in the remainder of this paper, while also relevant to other research approaches, will be presented primarily in the context of clinical trials.

There is increasing awareness of a need to address problems with research wastage and reproducibility in veterinary research; those issues are described in detail elsewhere (1, 3, 4). However, appropriate study designs and methods that address relevant research questions with well-defined outcomes valued by decision-makers are critical areas to be addressed in order to minimize research wastage and maximize value (1, 3). Therefore, the objective of this paper is to discuss the need and opportunities to define research outcomes that are relevant to stakeholders and to design studies that are driven directly by those defined outcomes.

## 2 Aligning outcomes with stakeholder values—what do they need for decision-making?

While stakeholder’s needs and input can have a tremendous impact on research relevance and value, the mechanism to engage stakeholders or understand their values can be as diverse as the spectrum of veterinary research. Broadly speaking, applied veterinary research can span from the “micro level,” where the end-user is a clinician or their clients, to the “macro level” in which decision-making can impact policy or a much larger population of animals or society (5). However, the critical question a researcher might ask is—what do “they” need for decision-making?

In some cases, the stakeholder(s) may indicate that decision-making simply requires a single key operational outcome that differs enough among intervention groups to be statistically significant and of a magnitude that is relevant clinically or economically for example. However, if decision-making is more complex, the stakeholder(s) may need research that demonstrates the impacts of an intervention on multiple conceptual outcome areas, each with key operational outcome measures. As an example, in Horton et al. (6), a stakeholder (cattle producer) was directly involved in a clinical trial to evaluate interventions for bovine respiratory disease with the primary goal of exploring options to reduce antimicrobial use. While antimicrobial use was the primary conceptual outcome, decision-making required other conceptual outcomes that also could be impacted by changes in antimicrobial use. Therefore, the study was designed with a primary operational outcome related to the number of antimicrobial doses, but also included other operational outcomes within the conceptual outcome areas of animal well-being, protein (beef) production, economics, and environmental impacts to address the stakeholder’s decision-making needs (5). Although multiple outcomes were used, these were defined *a priori* which is important in the context of appropriate study design, analysis, and reporting (1, 3, 7).

Given the scope and diversity of topics addressed by veterinary researchers, the type of research that stakeholders need also may vary tremendously. Thus, there can be no one standard process for gathering input from stakeholders before research is initiated. Depending on the research topic and scope, forming advisory groups, surveying content experts, connecting with professional networks, initiating stakeholder surveys, or other approaches may be beneficial and necessary for collecting *a priori* information on the needs of stakeholders. Regardless of the mechanism, it is critical to engage stakeholders early in the research process and understand their values in order to maximize the relevance and value of research. A challenging, but critically important component is that the stakeholder(s) and researcher(s) define the primary outcome measures needed for decision-making as these primary outcomes directly impact the research design (1).

## 3 Primary outcomes—designing, analyzing and reporting research accordingly

For all studies, the primary outcome(s) should be the outcome that is most relevant for decision-making by the target audience, and should be defined, reported, and used for the study design, including for calculating the necessary sample size to detect a meaningful difference in the magnitude of that primary outcome (1). By defining primary versus secondary outcomes, the end-user of the research should be able to determine which outcomes the study was powered to detect meaningful, statistically significant differences (primary) versus outcomes (secondary) not specifically used for the study design (1). The way in which a difference in the primary outcome will be considered meaningfully relevant should be defined with stakeholder input, and may be considered in terms that are related to any measure that stakeholders consider relevant to the issue of study, for instance an economic endpoint or one related to quality of life. This process of defining what difference (or lack thereof) in outcome that is meaningful to stakeholders should be followed regardless of the study design, or study purpose, even though the context differs for superiority, noninferiority, or equivalence studies (1).

Despite the importance of defining and reporting primary outcomes, implementation for applied veterinary research studies is generally poor. A scoping review of feedlot trials that included an economic outcome domain found that the primary outcome was stated in only 36% (41/113) of the trials (7). Similarly, of 91 dairy cattle trials published in 2017 with more than one outcome, the primary outcome was only identified in only 4 trials (8). In other reviews of veterinary literature, the reporting of primary outcomes was also low, and much lower compared to reports from human medical journals (1).

Failure to define and report primary outcomes, and appropriately design and analyze studies accordingly, can lead to several problems with research validity, reproducibility, and wastage that have been discussed in more detail elsewhere (1, 3, 7). Three of four major reasons for research wastage– addressing research questions that are not relevant, inadequate study design and methods, and biased or unusable results (1)—may be directly affected by poorly selected or defined outcomes. The lack of consistency in selection and reporting of outcomes also limits the ability to extend the value of individual studies through research synthesis methods (1).

The lack of appropriate selection, definition, and measurement of outcomes in designing, analyzing, and interpreting research studies can lead to bias and error (1, 3, 7). While errors are bound to happen even with well executed research and statistical methods (3), the traditional types of statistical errors—namely, type I and type II errors—can be inflated in studies that fail to avoid or address multiplicity in outcomes or fail to ensure adequate replication in relation to relevant differences in the primary outcome(s). Setting a type I error rate (α), of 5% for example, relates to a hypothesis test for a single outcome, and when multiple outcomes are analyzed (independently), the probability of type I errors can be quite large (1, 3). This problem can be further exacerbated—leading to bias—when reporting only the statistically significant results (1) or when only the statistically significant results are used for calculating some composite outcome(s), which involves combining multiple related outcomes into a single measure. As an example of the latter, consider that data are collected for multiple outcomes from cattle health and production data, but only those with statistically significant differences are used to calculate (*post hoc*) a composite economic outcome (7). This approach assumes that the variables omitted from the composite outcome are completely unaffected by the intervention which is a very strong, and often unrealistic assumption. In reality, the intervention may affect the omitted variables, but the study was underpowered to detect those differences so the composite variable is incorrect. Further, if there are no operational outcomes directly addressing the primary outcome domain (economics for example), but multiple surrogate operational outcomes are used based on results of hypothesis testing, type II errors also may occur (3, 7). Thus, in the context of the outcome domain of most relevance to the stakeholder, the results may be biased, and affected by some unknown combination of both type I and type II errors.

## 4 Discussion—outcomes research and potential solutions

The diversity and complexity of stakeholders and topics seem to make the provision of a standardized solution unrealistic for veterinary research as a whole. However, veterinary researchers can find guidance and potential solutions by looking to existing reporting guidelines for veterinary research or to other discipline areas such as those used for outcomes research, human health, and social sciences. For examples, recommendations for reporting outcomes for trials in pets (PETSORT) and livestock (REFLECT) are directly relevant and excellent resources (9–12). Reporting guidelines from other discipline areas, such as CHEERS for reporting economic assessments in human health studies, also are useful resources that have been used by veterinary researchers (7). However, none of the reporting guidelines provides guidance on how to appropriately identify and prioritize outcomes relative to stakeholder values.

A review on maximizing value and minimizing wastage in veterinary clinical trial research provides an excellent discussion on selection and reporting of outcomes (1). Among other topics, the authors discuss that one potential solution to improve consistency of outcome measures and reporting is the creation of core outcome sets, which represent an agreed upon minimum set of outcomes that should be reported in a specific topic area (1). The rationale and development of core outcome sets have been discussed in more detail elsewhere, but this approach has been applied in human healthcare much more frequently than in veterinary medicine (1). The ISPOR, a professional society for health economics and outcomes research developed nearly 30 years ago for human healthcare decision-making, has recently included animal- and one-health topics (4), and also provides resources including standards for health economics and outcomes research (13).

Two recent peer-reviewed reports on the relevance, value, and potential impacts of outcomes research in animal health and veterinary medicine provide excellent discussion of this discipline area (4, 14). While outcomes research principles are well-established in human medicine, their formal application in animal health and veterinary medicine are relatively new. The relevance here is that outcomes research explicitly focuses on defining both the potential effectiveness of an intervention (or policy) and the values of the stakeholders or research end-users (5). Thus, regardless of the domain, researchers prioritize outcomes that are valid and relevant to the end-users to maximize research value and to enable appropriate decision-making. That, in fact, should be the goal of researchers—to ensure validity throughout the research process, while also ensuring resulting outcomes appropriately inform and enable evidence- or science-based decision-making by the end-users.

## Data availability statement

The original contributions presented in the study are included in the article/supplementary material, further inquiries can be directed to the corresponding author.

## Author contributions

DR: Conceptualization, Writing – original draft. JS: Conceptualization, Writing – review & editing. AO’C: Conceptualization, Writing – review & editing. AR: Conceptualization, Writing – review & editing.
